# The association between blood pressure control in women during pregnancy and adverse perinatal outcomes: the TMM BirThree Cohort Study

**DOI:** 10.1038/s41440-023-01570-x

**Published:** 2024-01-18

**Authors:** Mami Ishikuro, Taku Obara, Keiko Murakami, Fumihiko Ueno, Aoi Noda, Tomomi Onuma, Masahiro Kikuya, Hirohito Metoki, Shinichi Kuriyama

**Affiliations:** 1 Tohoku Medical Megabank Organization, Tohoku University, 2-1 Seiryo-machi, Aoba-ku, Sendai, Miyagi 980-8573 Japan; 2 Tohoku University Graduate School of Medicine, 2-1 Seiryo-machi, Aoba-ku, Sendai, Miyagi 980-8575 Japan; 3 Tohoku University Hospital, 1-1 Seiryo-machi, Aoba-ku, Sendai, Miyagi 980-8574 Japan; 4 Teikyo University School of Medicine, 2-11-1 Kaga, Itabashi-ku, Tokyo 173-8605 Japan; 5 Faculty of Medicine, Tohoku Medical and Pharmaceutical University, 1-15-1, Fukumuro, Miyagino-ku, Sendai, Miyagi 983-8536 Japan; 6 International Research Institute of Disaster Science, Tohoku University, 2-1 Seiryo-machi, Aoba-ku, Sendai, Miyagi 980-8573 Japan

**Keywords:** Blood pressure control, Pregnancy, Adverse perinatal outcomes

## Abstract

Blood pressure (BP) control in pregnancy is essential to prevent adverse outcomes. However, BP levels for hypertension treatment are inconsistent among various guidelines. This study investigated the association between BP control and adverse perinatal outcomes. A total of 18,155 mother-offspring pairs were classified into four groups according to BP after 20 gestational weeks: normal BP (<140/90 mmHg without antihypertensive drugs), high BP (≥140/90 mmHg without antihypertensive drugs), controlled BP (<140/90 mmHg with antihypertensive drugs), and uncontrolled BP (≥140/90 mmHg with antihypertensive drugs). The prevalence of small for gestational age was 1,087/17,476 offspring in normal BP, 78/604 in high BP, 5/42 in controlled BP, and 7/33 in uncontrolled BP. Compared to normal BP, adjusted odds ratios (ORs) (95% confidence intervals (CIs)) were 1.76 (1.32–2.35) for high BP, 2.08 (0.79–5.50) for controlled BP, and 2.34 (0.94–5.85) for uncontrolled BP (multiple logistic regression analysis). Similarly, the adjusted ORs (95% CIs) were 1.80 (1.35–2.41), 3.42 (1.35–8.63), and 5.10 (1.93–13.45) for high, controlled, and uncontrolled BPs for low birth weight, respectively; 1.99 (1.48–2.68), 2.70 (1.12–6.50), and 6.53 (3.09–13.82) for high, controlled, and uncontrolled BPs for preterm birth, respectively; 1.64 (1.19–2.24), 2.17 (0.88–5.38), and 2.12 (0.80–5.65) for high, controlled, and uncontrolled BPs for admission to the Neonatal Intensive Care Unit or Growing Care Unit, respectively; and 1.17 (0.70–1.95), 2.23 (0.65–7.68), and 0.91 (0.20–4.16) for high, controlled, and uncontrolled BPs for 1-min Apgar score < 7, respectively. BP ≥ 140/90 mmHg might be taken care for preventing various adverse perinatal outcomes.

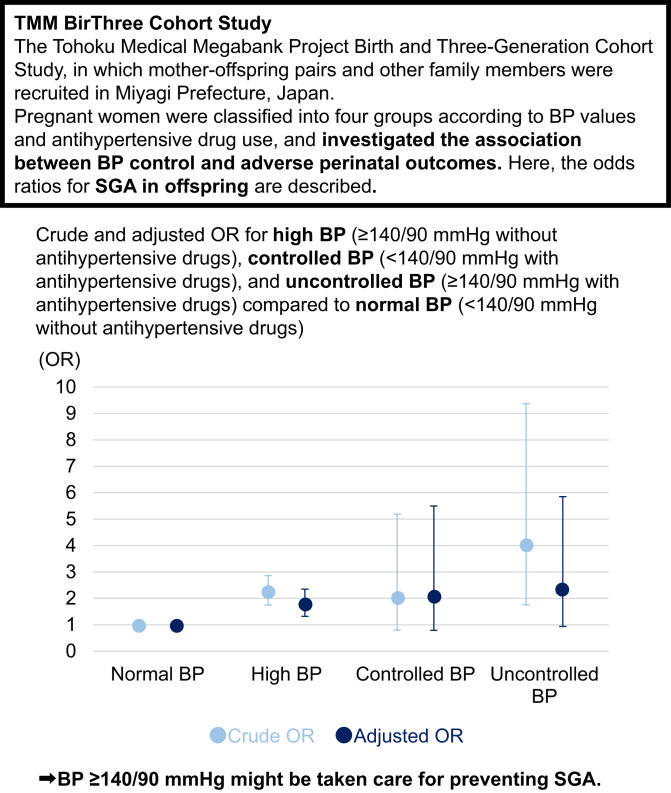

## Introduction

Hypertensive disorders of pregnancy increase the risk of many adverse perinatal outcomes, such as perinatal and fetal deaths, small for gestational age (SGA), low birth weight, and preterm birth [1]. Blood pressure (BP) control is essential to prevent such adverse outcomes. In Japan, the contraindication of amlodipine and nifedipine in pregnant women has recently been removed, which increases the spectrum of therapies for BP control during pregnancy. However, BP levels for hypertension treatment are inconsistent among various guidelines [2–4]. SGA is a long-term risk factor for the offspring’s prognosis. Further evidence of the effect of BP control during pregnancy on perinatal outcomes is required. The objective of this study was to investigate the association between BP control in women and SGA, low birth weight, preterm birth, neonatal admission, and Apgar score in their offspring.

Point of view

- Clinical relevance
  Pregnant woman’s blood pressure ≥140/90 mmHg might be taken care for preventing various adverse perinatal outcomes.
- Future direction
  The efficacy of blood pressure control with antihypertensive drug use during pregnancy for both maternal and child adverse outcomes should be confirmed in randomized controlled trials.
- Consideration for the Asian population
  The prevalence of hypertension in pregnancy and its severity is different among ethnicities. More evidence followed the trend of antihypertensive drug use in Japan and Asia is necessary.

## Methods

This study formed a part of the Tohoku Medical Megabank Project Birth and Three-Generation Cohort Study (TMM BirThree Cohort Study), in which 23,730 mother-offspring pairs and other family members were recruited in Miyagi Prefecture, Japan [5]. The protocol of the TMM BirThree Cohort Study was approved by the Tohoku Medical Megabank Organization’s internal review board (approval no.: 2013-1-103-1), and written informed consent was obtained from all the participants.

For this study, we excluded women who withdrew from the study; those who had multiple pregnancies, abortions, or stillbirths; and those for whom data on medication use, BP, parity, gestational weeks at birth, child birth weight, child sex, and any other covariates were missing. Pregnant women were classified into four groups. The “normal BP” group comprised women who did not use antihypertensive drugs and had <140 mmHg systolic BP and <90 mmHg diastolic BP after 20 gestational weeks during pregnancy. The “high BP” group comprised women who did not use antihypertensive drugs and had ≥140 mmHg systolic BP or ≥90 mmHg diastolic BP at least twice after 20 gestational weeks during pregnancy. The “controlled BP” group comprised women who used antihypertensive drugs during pregnancy and had <140 mmHg systolic BP and <90 mmHg diastolic BP after 20 gestational weeks during pregnancy. The “uncontrolled BP” group comprised women who used antihypertensive drugs during pregnancy and had ≥140 mmHg systolic BP or ≥90 mmHg diastolic BP at least twice after 20 gestational weeks during pregnancy. BP was extracted from medical charts at participating obstetrical hospitals and clinics in Miyagi Prefecture by genome medical research coordinators [6]. The use of specific antihypertensive drugs were determined using self-reported questionnaires in the first and second trimesters, according to the Japanese Society of Hypertension Guidelines for the Management of Hypertension and a previous study [7, 8]. The overall antihypertensive drug use in the TMM BirThree Cohort Study was previously described [9]. BP values were collected from medical charts.

SGA was calculated on the basis of maternal parity, gestational age at birth, birth weight, and sex, all collected from medical charts. SGA was defined as birth weight in the 1st to 10th percentiles [10, 11]. Low birth weight was defined as children whose birth weight were less than 2500 g. Preterm birth was defined as delivery less than 37 gestational weeks. Neonatal admission (admission to the pediatric ward, admission to the Neonatal Intensive Care Unit (NICU) or Growing Care Unit (GCU)) and Apgar score at 1 and 5 min were collected from medical charts at birth by genome medical research coordinators. An adverse outcome based on Apgar score <7 was set.

Characteristics of participants were obtained from self-reported questionnaires and medical charts. Each item was compared by analysis of variance, chi-squared test, and Fisher’s exact test, appropriately. Multiple logistic regression analyses were performed to determine the association between BP and SGA, low birth weight, preterm birth, neonatal admission and 1-min Apgar score <7. About 5-min Apgar score, its prevalence was only described because the model did not fit. In the model, we adjusted for maternal age at delivery, maternal pre-pregnancy body mass index (BMI), maternal smoking, preterm birth (except as an outcome), and maternal diastolic BP level after 20 gestational weeks to investigate the relationship between the management status in the late pregnancy period and perinatal outcome as in model 1. In model 2, placental weight was included additionally. Statistical analyses were performed using SAS (version 9.4; SAS Institute Inc., Cary, NC, USA).

## Results

In total, 18,155 mother-offspring pairs were eligible for this study. Among them, 17,476 women had normal BP, 604 had high BP, 42 had controlled BP, and 33 had uncontrolled BP. Women who used antihypertensive drugs were older and had a higher pre-pregnancy BMI than those who did not (Table 1). Less than 1% of women in each of the normal and high BP groups used antihypertensive drugs in the last year before pregnancy, while about 57 and 45% in the controlled and uncontrolled BP groups did, respectively. About 71% of women in the controlled BP group and 70% of those in the uncontrolled BP group used antihypertensive drugs during the first trimester, and about 83% of those in the controlled BP group and 82% of those in the uncontrolled BP group used antihypertensive drugs in the second trimester.

**Table 1:** Basic characteristics

	Normal BP (*n* = 17,476)	High BP (*n* = 604)	Controlled BP (*n* = 42)	Uncontrolled BP (*n* = 33)	*P* value
Maternal age (years)	31.8 ± 4.9	32.9 ± 5.2	35.0 ± 5.3	35.9 ± 5.7	<0.0001
Maternal pre-pregnancy BMI (kg/m^2^)	21.3 ± 3.3	24.0 ± 5.1	26.0 ± 7.2	24.9 ± 5.5	<0.0001
Maternal smoking					0.1
Never	10,650 (60.9)	345 (57.1)	24 (57.1)	17 (51.5)	
Stop smoking before/after pregnancy	6447 (36.9)	238 (39.4)	18 (42.9)	16 (48.5)	
Current	379 (2.2)	21 (3.5)	0	0	
Maternal alcohol consumption					0.2
Never	7949 (45.5)	302 (50.0)	18 (42.9)	12 (36.4)	
Stop drinking	6045 (34.6)	195 (32.3)	17 (40.5)	18 (54.6)	
Drinking	3444 (19.7)	105 (17.4)	7 (16.7)	3 (9.1)	
Missing	38 (0.2)	2 (0.3)	0	0	
Maternal systolic BP at 1st trimester (mmHg)	110.7 ± 11.3	127.3 ± 13.6	125.9 ± 16.7	138.0 ± 14.2	<0.0001
Maternal diastolic BP at 1st trimester (mmHg)	64.3 ± 8.9	77.1 ± 10.6	80.2 ± 12.1	86.2 ± 9.6	<0.0001
Maternal glucose level at 1st trimester (mg/dl)	84.5 ± 15.0	87.1 ± 13.1	93.0 ± 19.2	84.3 ± 10.6	0.0003
Proteinuria at 1st trimester					<0.0001
± or less	16,183 (92.6)	530 (87.8)	34 (81.0)	24 (72.7)	
+ 1 or more at least once	936 (5.4)	49 (8.1)	3 (7.1)	2 (6.1)	
Missing	357 (2.0)	25 (4.1)	5 (11.9)	7 (21.2)	
Maternal systolic BP after 20 gestational weeks (mmHg)	110.5 ± 8.3	130.3 ± 8.2	120.7 ± 8.7	135.3 ± 5.7	<0.0001
Maternal diastolic BP after 20 gestational weeks (mmHg)	63.6 ± 6.8	79.7 ± 7.2	73.3 ± 7.2	85.6 ± 6.9	<0.0001
Maternal antihypertensive drug use					
Last year before pregnancy	32 (0.2)	6 (1.0)	24 (57.1)	15 (45.5)	<0.0001
1st trimester	0	0	30 (71.4)	23 (69.7)	<0.0001
2nd trimester	0	0	35 (83.3)	27 (81.8)	<0.0001

*BMI* body mass index, *BP* blood pressure

Data are displayed as counts (percentages) or means ± standard deviations, as appropriate

Glucose level was available for 11,416 pregnant women

SGA was observed in 1087 (6.2%) offspring in the normal BP group, 78 (12.9%) in the high BP group, 5 (11.9%) in the controlled BP group, and 7 (21.2%) in the uncontrolled BP group (Table 2). Compared to normal BP, crude odds ratios (ORs) (95% confidence intervals [CIs]) and adjusted ORs (95% CIs) in model 1 were 2.24 (1.75–2.86) and 1.76 (1.32–2.35) for high BP, respectively, 2.04 (0.80–5.19) and 2.08 (0.79–5.50) for controlled BP, respectively, and 4.06 (1.76–9.37) and 2.34 (0.94–5.85) for uncontrolled BP, respectively. In model 2, SGA was observed in 971/15,813 (6.1%) children in the normal BP group, 71/557 (12.8%) children in the high BP group, 5/40 (12.5%) children in the controlled BP group, and 7/31 (22.6%) children in the uncontrolled BP group. Only high BP was associated with a higher prevalence of SGA compared to normal BP.

**Table 2:** Prevalence and odds ratios for perinatal outcomes

Perinatal outcomes		Normal BP	High BP	Controlled BP	Uncontrolled BP
SGA	Prevalence (%)	1,087/17,476 (6.2)	78/604 (12.9)	5/42 (11.9)	7/33 (21.2)
	Crude OR [95%CI]	ref	2.24 [1.75–2.86]	2.04 [0.80–5.19]	4.06 [1.76–9.37]
	Model 1 adjusted OR [95%CI]	ref	1.76 [1.32–2.35]	2.08 [0.79–5.50]	2.34 [0.94–5.85]
	Model 2 adjusted OR [95%CI]	ref	1.53 [1.10–2.13]	1.46 [0.50–4.28]	1.12 [0.37–3.40]
Low birth weight	Prevalence (%)	1,325/17,476 (7.6)	114/604 (18.9)	10/42 (23.8)	16/33 (48.5)
	Crude OR [95%CI]	ref	2.84 [2.30–3.50]	3.81 [1.87–7.77]	11.47 [5.78–22.75]
	Model 1 adjusted OR [95%CI]	ref	1.80 [1.35–2.41]	3.42 [1.35–8.63]	5.10 [1.93–13.45]
	Model 2 adjusted OR [95%CI]	ref	1.66 [1.19–2.32]	2.68 [1.01–7.10]	3.44 [1.03–11.56]
Preterm birth	Prevalence (%)	798/17,476 (4.6)	75/604 (12.4)	6/42 (14.3)	12/33 (36.4)
	Crude OR [95%CI]	ref	2.96 [2.30–3.81]	3.48 [1.46–8.29]	11.94 [5.86–24.36]
	Model 1 adjusted OR [95%CI]	ref	1.99 [1.48–2.68]	2.70 [1.12–6.50]	6.53 [3.09–13.82]
	Model 2 adjusted OR [95%CI]	ref	1.95 [1.43–2.66]	2.19 [0.89–5.40]	5.02 [2.21–11.40]
Neonatal admission	Neonatal ward (%)	986/17,476 (5.6)	56/604 (9.3)	0/42 (0)	4/33 (12.1)
	Crude OR [95%CI]	ref	1.94 [1.46–2.58]	–	3.50 [1.18–10.36]
	Model 1 adjusted OR [95%CI]	ref	1.87 [1.35–2.59]	–	2.48 [0.79–7.80]
	Model 2 adjusted OR [95%CI]	ref	1.87 [1.35–2.61]	–	2.36 [0.73–7.62]
	NICU or GCU (%)	969/17,476 (5.5)	93/604 (15.4)	9/42 (21.4)	11/33 (33.3)
	Crude OR [95%CI]	ref	3.27 [2.60–4.13]	4.37 [2.08–9.16]	9.79 [4.61–20.78]
	Model 1 adjusted OR [95%CI]	ref	1.64 [1.19–2.24]	2.17 [0.88–5.38]	2.12 [0.80–5.65]
	Model 2 adjusted OR [95%CI]	ref	1.65 [1.20–2.28]	1.89 [0.76–4.68]	1.89 [0.68–5.24]
1-min Apgar score <7	Prevalence (%)	334/16,261 (2.1)	23/567 (4.1)	3/41 (7.3)	2/32 (6.3)
	Crude OR [95%CI]	ref	2.02 [1.31–3.10]	3.77 [1.16–12.26]	3.18 [0.76–13.36]
	Model 1 adjusted OR [95%CI]	ref	1.17 [0.70–1.95]	2.23 [0.65–7.68]	0.91 [0.20–4.16]
	Model 2 adjusted OR [95%CI]	ref	1.29 [0.76–2.18]	2.33 [0.67–8.05]	0.94 [0.20–4.33]
5-min Apgar score <7	Prevalence (%)	97/15,952 (0.6)	5/560 (0.9)	0/41 (0)	0/32 (0)
	Crude OR [95%CI]	–	–	–	–
	Model 1 adjusted OR [95%CI]	–	–	–	–
	Model 2 adjusted OR [95%CI]	–	–	–	–

*SGA* small for gestational age, *OR* odds ratio, *CI* confidence interval, *NICU* Neonatal Intensive Care Unit, *GCU* Growing Care Unit, *BP* blood pressure

Model 1 was adjusted for maternal age at delivery, pre-pregnancy body mass index, maternal smoking, preterm birth (except as an outcome), and maternal diastolic BP level after 20 gestational weeks

Model 2 was adjusted for placental weight in addition to model 1. The total number of participants in model 2 was 16,441 each for SGA, low birth weight, preterm birth, and neonatal admission and 16,375 for 1-min Apgar score <7

Similarly, in model 1, the adjusted ORs (95% CIs) were 1.80 (1.35–2.41), 3.42 (1.35–8.63), and 5.10 (1.93–13.45) for high, controlled, and uncontrolled BPs for low birth weight, respectively; 1.99 (1.48–2.68), 2.70 (1.12–6.50), and 6.53 (3.09–13.82) for high, controlled, and uncontrolled BPs for preterm birth, respectively. In model 2, the adjusted ORs (95% CIs) were 1.66 (1.19–2.32), 2.68 (1.01–7.10), and 3.44 (1.03–11.56) for high, controlled, and uncontrolled BPs for low birth weight, respectively, and 1.95 (1.43–2.66), 2.19 (0.89–5.40), and 5.02 (2.21–11.40) for high, controlled, and uncontrolled BPs for preterm birth, respectively.

The prevalence of the admission to the neonatal ward for normal, high, controlled, and uncontrolled BP groups were 6.1%, 9.9%, 0%, and 12.9%, and the prevalence of the admission to the NICU/GCU were 6.0%, 16.7%, 22.5%, and 35.5%. Compared to normal BP, adjusted ORs (95% CIs) for the admission to the neonatal ward were 1.87 (1.35–2.59) for high BP and 2.48 (0.79–7.80) for uncontrolled BP in model 1. Adjusted ORs (95% CIs) for the admission to the NICU/GCU were 1.64 (1.19–2.24) for high BP, 2.17 (0.88–5.38) for controlled BP, and 2.12 (0.80–5.65) for uncontrolled BP in model 1.

As for Apgar score, 16,901 and 16,585 children were available for the score at 1 and 5 min. Compared to normal BP, adjusted ORs (95% CIs) for 1-min Apgar score < 7 were 1.17 (0.70–1.95) for high BP, 2.23 (0.65–7.68) for controlled BP, and 0.91 (0.20–4.16) for uncontrolled BP in model 1.

## Discussion

The present study revealed that an uncontrolled BP despite antihypertensive drug use during pregnancy is associated with a higher prevalence of low birth weight, preterm birth, and neonatal admission than a normal BP during pregnancy in model 1. The OR of uncontrolled BP for SGA was also high. SGA partially share the characteristics with low birth weight and preterm birth. Neonatal admission is also led by those adverse conditions; therefore, the results were similar among those adverse perinatal outcomes. Severe hypertension is recognized as a condition that must be treated during pregnancy for better maternal outcomes, while the risk of SGA has also been discussed [12–14]. Among chronic hypertension, a previous study of systematic review and meta-analysis reported no significant difference in odds between antihypertensive treated and untreated women for perinatal outcomes except for SGA [15]. More recently, Tita et al. reported that active treatment of pregnant women for mild hypertension (<160/100 mmHg), achieving a BP of <140/90 mmHg, did not increase the prevalence of SGA among their offspring compared to that of the offspring of pregnant women not receiving such treatment [16]. There is an update meta-analysis of randomized controlled trial that antihypertensive treatment was associated with better outcomes in maternal complications and neonatal mortality for pregnant women with mild hypertension (140–159/90–109 mmHg), without increased a chance of SGA [17]. Our results that the risk of adverse perinatal outcomes was high in uncontrolled BP might support those findings that BP during pregnancy should be controlled to <140/90 mmHg. However, the efficacy of antihypertensive drug use should be confirmed in randomized controlled trials.

In this study, high BP was associated with a higher prevalence of SGA, while controlled BP and the prevalence of SGA were not associated. Furthermore, the ORs were lower for both controlled and uncontrolled BPs than for high BP in model 2. This result may be due to the low statistical power by the small number of participants; however, confounding by antihypertensive drug use might be also considered. Block-Abraham et al. showed that regardless of BP level at enrollment, SGA was concentrated in women with higher average BP in the second and third trimester, and BP improvement throughout pregnancy decreased preeclampsia rate without increasing SGA [18]. Our results suggest that both placental growth and BP control using antihypertensive drugs after 20 weeks might be important for preventing SGA in children. About low birth weight and preterm birth, the ORs among controlled BP group was slightly higher than those among high BP group when those ORs were compared to normal BP group. This might express that more severe women tend to be treated because this is an observational study. On the other hand, this might also indicate the importance of treatment. None of the participants who had 5-min Apgar score <7 was observed in controlled and uncontrolled BP. However, the logistic regression model did not fit. Further studies are needed to determine whether BP controlled to <140/90 mmHg reduces the risk of adverse perinatal outcomes.

Although we assessed the use of antihypertensive drugs during pregnancy and investigated the prevalence of adverse perinatal outcomes according to BP control status, with detailed maternal and offspring information obtained from a birth cohort study, this study has several limitations. First, we did not collect information about antihypertensive drug use in the third trimester, the trimester of pregnancy in which the prevalence of antihypertensive drug prescription is the highest in Japan [19]; therefore, we might have misclassified the mother-offspring pairs in this study. Second, even though the TMM BirThree Cohort Study contained a large number of mother-offspring pairs, not many of the pregnant women used antihypertensive drugs, resulting in a wide 95% CI. Some of the adverse perinatal outcomes, such as congenital abnormality was not available in this study. Morphologic abnormality was previously assessed among amlodipine use in the first trimester, other antihypertensive drug use, and no treatment Japanese women, and concluded that the ORs were not different among them [20]. This study also could not investigate differences among type of hypertension in pregnancy and early/late onset. In addition, because of lack of information or availability, this study could not consider blood glucose level, renal function, congenital infections, or alcohol consumption which might affect some of the adverse perinatal outcomes. In Japan, a large clinical database with detailed information on mothers and their offspring is lacking. Meta-analyses with other birth cohort studies, with adjustment for confounders, should improve the reliability of the results and allow the formulation of recommendations for best clinical practice.

## Asian perspectives

The prevalence of hypertension in pregnancy and its severity is different among ethnicities. The recent report by Leonard et al. described that the prevalence of chronic hypertension during pregnancy in Asian is 1.6% while 2.5% in American Indian or Alaska Native, 5.1% in Black, 1.5% in Latino, 2.9% in Native Hawaiian or Other Pacific Islander, 2.0% in White, and 2.3% in Multiracial or Other people [21]. Leonard et al. also mentioned that risk ratio for preeclampsia with severe features or eclampsia is high in Asian. Also, antihypertensive drug use among pregnant women is low in Japan and Korea compared to other countries [19], reflecting the prevalence of hypertension in pregnancy. Although the proportion of antihypertensive drug prescriptions among pregnant women has not been changed from the year 2013 to 2020, the trend of selection for each antihypertensive drug has been changed [22]. More evidence followed the trend of antihypertensive drugs in Japan and Asia is necessary.

## Conclusion

The BP value is an important consideration for the prevention of adverse perinatal outcomes. In particular, our results that both uncontrolled and high BP were associated with an increased risk of adverse perinatal outcomes suggest that the pregnant woman’s BP ≥ 140/90 mmHg might be taken care for preventing various adverse perinatal outcomes.
